# Real-World Comparative Effectiveness of Ferric Derisomaltose Versus Conventional Iron Treatment in Postpartum Hemorrhage-Related Anemia

**DOI:** 10.7759/cureus.93490

**Published:** 2025-09-29

**Authors:** Takuya Misugi, Emi Nakamoto, Yasushi Kurihara, Kohei Kitada, Akihiro Hamuro, Mie Tahara, Akemi Nakano, Daisuke Tachibana

**Affiliations:** 1 Obstetrics and Gynecology, Osaka Metropolitan University Graduate School of Medicine, Osaka, JPN

**Keywords:** clinical effectiveness, ferric derisomaltose, ferritin, intravenous iron therapy, iron deficiency anemia, iron supplementation, postpartum anemia, postpartum hemorrhage, real-world evidence

## Abstract

Background: Postpartum hemorrhage (PPH) can lead to acute iron deficiency anemia, negatively affecting maternal health and infant care by contributing to fatigue, depression, and difficulties with breastfeeding. There is a clinical need for rapid and effective iron supplementation.

Objective: This study aimed to evaluate the real-world effectiveness of ferric derisomaltose (FDI), which allows for single high-dose intravenous administration, in comparison with conventional treatment combining intravenous and oral iron administration.

Methods: This retrospective observational study included postpartum women with hemoglobin (Hb) levels <8 g/dL within three days after delivery between April 2023 and April 2025. Patients in the FDI group received a single 1000 mg intravenous dose of FDI, while the conventional treatment group received intravenous saccharated ferric oxide and oral sodium ferrous citrate. Hematologic parameters (Hb, serum iron, total iron-binding capacity, ferritin, reticulocyte count, and serum phosphate) were evaluated at two weeks and one month postpartum.

Results: A total of 91 patients met the inclusion criteria (38 in the conventional treatment group and 53 in the FDI group). Hb levels were significantly higher and anemia rates significantly lower in the FDI group at both two weeks and one month postpartum. Serum iron and ferritin levels were also significantly higher in the FDI group. No cases of hypophosphatemia were observed in either group.

Conclusion: Single-dose intravenous FDI demonstrated earlier and more effective correction of postpartum anemia compared with conventional treatment, with a favorable safety profile. FDI may represent a strong treatment option in real-world clinical practice.

## Introduction

Postpartum hemorrhage (PPH) is one of the most serious complications following delivery and is a major cause of acute iron deficiency anemia. Postpartum anemia has been linked to maternal fatigue, impaired concentration [1], depressive symptoms [2-5], and challenges in breastfeeding and infant care [6,7], potentially affecting both maternal and infant well-being. Timely and effective iron supplementation is thus crucial in the early postpartum period.

Ferric derisomaltose (FDI), a newer intravenous iron formulation, is composed of a stable complex of polynuclear iron(III)-hydroxide tightly bound to an isomaltoside matrix (~1000 Da), resulting in the minimal release of free iron [8]. This allows safe, high-dose, single-administration intravenous treatment, an advantage over older iron formulations that require multiple doses and significant medical resource allocation. Oral iron, while non-invasive, suffers from low bioavailability and poor patient adherence, leading to variable treatment outcomes.

While the efficacy of FDI has been demonstrated in randomized controlled trials (RCTs) conducted primarily overseas [9-12], a domestic phase III trial has also shown non-inferiority to existing intravenous iron treatments [13]. However, RCTs often use strict inclusion criteria that may not reflect real-world clinical settings, highlighting the need for complementary observational data.

This study aims to compare the real-world effectiveness of single-dose FDI versus combined intravenous and oral iron treatment in improving hemoglobin (Hb) levels among postpartum women with anemia caused by PPH.

## Materials and methods

Study design and setting

This was a single-center, retrospective observational study conducted at Osaka Metropolitan University Hospital, a tertiary care academic medical center located in Osaka, Japan. The study period spanned from April 2023 to April 2025. The study protocol was reviewed and approved by the Ethical Committee of Osaka Metropolitan University Graduate School of Medicine (approval number: 2023-075). All procedures were conducted in accordance with the ethical standards of the institutional and national research committee and with the 1964 Helsinki Declaration and its later amendments.

Participants and eligibility criteria

Eligible participants were postpartum women who delivered at the hospital during the study period and had an Hb level of less than 8 g/dL measured within the first three days following delivery. These women were identified through the hospital's electronic medical record system. Exclusion criteria included the following: (1) receipt of a red blood cell transfusion after delivery, as this could confound hematologic recovery, and (2) incomplete clinical or laboratory data preventing adequate analysis.

Patients were retrospectively categorized into one of the two treatment groups based on the clinical treatment they received: The FDI group included patients who were administered a single intravenous dose of 1000 mg FDI, diluted in 100 mL of normal saline and infused over 30 minutes. The traditional treatment group consisted of patients who received 80 mg/day of saccharated ferric oxide (SFO) intravenously for three consecutive days during hospitalization. These intravenous administrations were also performed by nursing staff under physician supervision. Following the intravenous injections, patients were prescribed oral sodium ferrous citrate at a dose of 500 mg/day, which they were instructed to take daily until their one-month postpartum outpatient visit.

Outcomes and measurements

The primary outcomes were hematological parameters evaluated at two weeks and one month postpartum, including Hb, serum iron, total iron-binding capacity (TIBC), serum phosphate, reticulocyte count, and serum ferritin (measured only at one month postpartum).

Blood samples were collected during the scheduled postpartum outpatient visits at two weeks and one month and analyzed by the hospital's central laboratory. Anemia was defined according to the World Health Organization criteria as Hb <11 g/dL. The proportion of patients classified as anemic at each follow-up point was compared between groups.

Additionally, perinatal characteristics such as maternal age, gestational age at delivery, mode of delivery, and neonatal outcomes were extracted to assess group comparability.

Statistical analysis

All statistical analyses were conducted using IBM SPSS Statistics for Windows, Version 21.0 (IBM Corp., Armonk, New York, United States). Continuous variables were expressed as medians with ranges and compared between groups using the Mann-Whitney U test, as the data did not follow a normal distribution. Categorical variables were presented as counts and percentages and assessed using chi-squared (χ²) tests.

All p-values were two-tailed, and values less than 0.05 were considered statistically significant. In accordance with best practices for non-parametric statistical comparisons, U-values for the Mann-Whitney U tests and χ² values for categorical comparisons were also reported in tables.

As this was a retrospective study, a formal sample size calculation was not performed a priori. However, a post hoc power analysis was conducted based on observed differences in Hb levels between groups at two weeks postpartum (FDI group: median 11.2 g/dL; traditional group: median 10.8 g/dL). With a sample size of 53 in the FDI group and 38 in the control group, this study had an estimated power of approximately 80% to detect a moderate effect size at a significance level of 0.05, assuming a Mann-Whitney U test.

## Results

**Figure 1:**
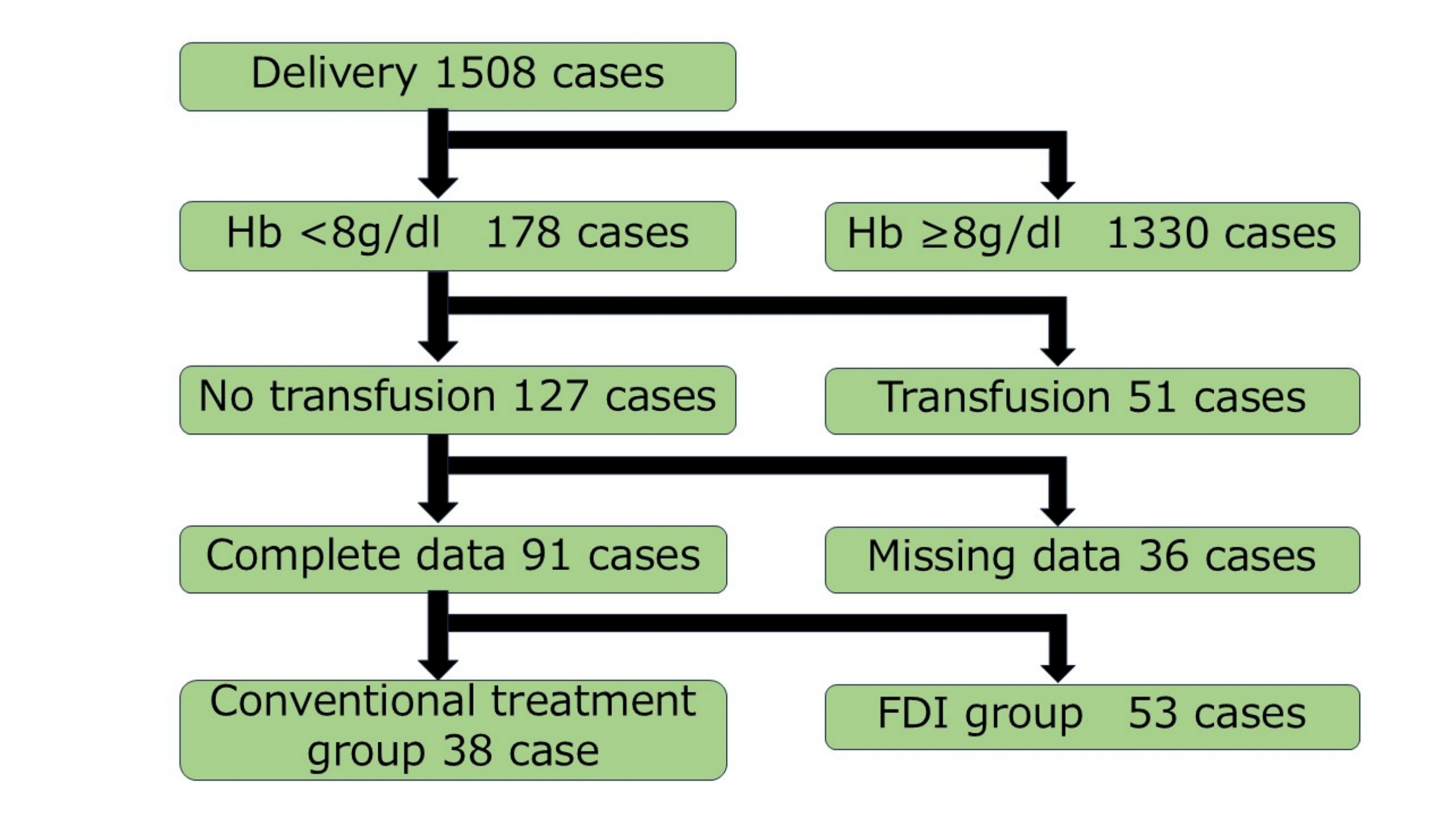
Flowchart of patient selection for analysis Flowchart showing the selection of 91 patients with postpartum anemia from 1,508 deliveries between April 2023 and April 2025. Patients were divided into the conventional therapy group (n=38) and the FDI group (n=53) after exclusions. Hb: hemoglobin; FDI: ferric derisomaltose

During the study period (April 2023 to April 2025), a total of 1,508 women delivered at our hospital. Among them, 178 patients had Hb levels <8 g/dL within three days postpartum. After excluding 51 patients who received blood transfusions and 36 with incomplete medical records, 91 patients were included in the final analysis (38 in the conventional treatment group and 53 in the FDI group) (Figure 1).

Patients' characteristics are summarized in Table 1. There were no significant differences between the two groups in terms of age, nulliparity, use of assisted reproductive technology, height, prepregnant body weight, or body mass index (BMI).

**Table 1:** Patients' characteristics Comparison of the baseline characteristics of patients with postpartum anemia in the traditional treatment group and the FDI group. Continuous variables are presented as medians (ranges) and were analyzed using the Mann-Whitney U test, with p-values marked by †. Categorical variables are presented as counts (%) and were analyzed using the chi-squared test, with p-values marked by ‡. A p-value of less than 0.05 was considered statistically significant. No significant differences were observed between groups in any variables. FDI: ferric derisomaltose; ART: assisted reproductive technology; BMI: body mass index

Variables	Conventional treatment group (n=38)	FDI group (n=53)	P-value
Age (years)	34 (24-45)	34 (25-43)	0.93 (U=978)^†^
Primipara (%)	24 (63.2)	33 (62.3)	0.93 (χ2=0.01)^‡^
ART (%)	18 (47.4)	26 (49.1)	0.87 (χ2=0.03)^‡^
Height (cm)	158 (140-173)	158 (145-175)	0.63 (U=930)^†^
Prepregnant body weight (kg)	55 (35-83)	53 (40-87)	0.44 (U=821)^†^
Prepregnant BMI (kg/m^2^)	21.8 (17.1-35.0)	21.3 (15.8-35.3)	0.52 (U=835)^†^

Perinatal outcomes are presented in Table 2. The rate of cesarean section (CS) was significantly higher in the conventional treatment group (76.3%, 29/38) compared to the FDI group (35.8%, 19/53) (p<0.01). However, there were no significant differences in blood loss volume at delivery or nadir postpartum Hb levels between the groups.

**Table 2:** Perinatal outcomes Comparison of perinatal outcomes between the traditional treatment group and the FDI group. Continuous variables are presented as medians (ranges) and were analyzed using the Mann-Whitney U test, with p-values marked by †. Categorical variables are presented as counts (%) and were analyzed using the chi-squared test, with p-values marked by ‡. A p-value of less than 0.05 was considered statistically significant. A significantly higher rate of CS was observed in the traditional treatment group compared to the FDI group. FDI: ferric derisomaltose; GA: gestational age; CS: cesarean section; Hb: hemoglobin

Variables	Traditional treatment group (n=38)	FDI group (n=53)	P-value
GA (days)	268 (180-292)	270 (192-290)	0.96 (U=982)^†^
CS (%)	29 (76.3)	19 (35.8)	<0.01 (Χ^2^=14.5)^‡^
Blood loss (mL)	1043	1145 (200-3175)	0.67 (U=937)^†^
Multiple pregnancy (%)	3 (7.9)	6 (11.3)	0.99 (Χ^2^=0.29)^‡^
Birth weight (g)	3125 (500-3745)	2960 (990-4260)	0.8 (U=957)^†^
Lowest postpartum Hb (g/dL)	7.5 (5.4-7.9)	7.3 (5.7-7.9)	0.88 (U=993)^†^

Hematological parameters at the two-week postpartum checkup are shown in Table 3. Median Hb levels were significantly higher in the FDI group (11.2 g/dL) than in the conventional treatment group (10.9 g/dL; p=0.03). Serum iron levels were also significantly higher in the FDI group, while TIBC was significantly lower (both p<0.05). No significant differences were found in reticulocyte counts between groups. The anemia rate (Hb <11 g/dL) was significantly lower in the FDI group (34%, 18/53) compared to the conventional treatment group (68.4%, 26/38; p<0.05).

**Table 3:** Hematological parameters at two weeks postpartum Comparison of hematological parameters two weeks postpartum between the traditional treatment group and the FDI group. Continuous variables are presented as medians (ranges) and were analyzed using the Mann-Whitney U test, with p-values marked by †. Categorical variables are presented as counts (%) and were analyzed using the chi-squared test, with p-values marked by ‡. A p-value of less than 0.05 was considered statistically significant. Hb and serum iron levels and TIBC showed statistically significant differences between the two groups: the FDI group exhibited higher Hb and serum iron levels and lower TIBC. The proportion of patients with anemia (Hb <11 g/dL) was also significantly lower in the FDI group. FDI: ferric derisomaltose; Hb: hemoglobin; TIBC: total iron-binding capacity

Variables	Traditional treatment group (n=38)	FDI group (n=53)	P-value
Days from delivery (days)	20 (12-26)	17 (10-26)	0.92 (U=987)^†^
Hb (g/dL)	10.9 (8.8-12.6)	11.2 (9.1-13.5)	0.03 (U=706)^†^
ΔHb (from baseline) (g/dL)	3.7 (1.3-5.2)	3.8 (2.5-6.2)	0.07 (U=770)^†^
Reticulocyte (‰)	29 (10-92)	33 (12-102)	0.45 (U=906)^†^
Serum phosphorus (mg/dL)	4 (2.9-5.2)	3.8 (2.7-4.8)	0.13 (U=737)^†^
Serum iron (mg/dL)	52 (14-143)	78 (10-150)	<0.01 (U=428)^†^
TIBC (μg/dL)	361 (244-41)	325 (251-437)	0.02 (U=776)^†^
Hb <11 g/dL (%)	26 (68.4)	18 (34)	<0.01 (Χ^2^＝4.1)^‡^

Hematological parameters at the one-month postpartum checkup are presented in Table 4. The FDI group had a significantly higher median Hb level (12.3 g/dL) than the conventional treatment group (11.7 g/dL; p<0.01). Serum iron was also higher in the FDI group (73 mg/dL vs. 60 mg/dL), as was serum ferritin (257 ng/mL vs. 79 ng/mL), with both differences being statistically significant (p<0.01). TIBC was significantly lower in the FDI group (275 μg/dL vs. 323 μg/dL; p<0.01). No significant differences were observed in serum phosphate levels or reticulocyte counts. Importantly, no cases of hypophosphatemia were observed in either group. Regarding anemia rates at one month, 18.4% (7/38) of patients in the conventional treatment group remained anemic (Hb <11 g/dL), whereas none of the patients (0/53) in the FDI group were anemic statistically significant difference (p<0.01).

**Table 4:** Hematological parameters at one month postpartum Comparison of hematological parameters at one month postpartum between the traditional treatment group and the FDI group. Continuous variables are presented as medians (ranges) and were analyzed using the Mann-Whitney U test, with p-values marked by †. Categorical variables are presented as counts (%) and were analyzed using the chi-squared test, with p-values marked by ‡. A p-value of less than 0.05 was considered statistically significant. Hb, serum iron, and ferritin levels were significantly higher in the FDI group, while TIBC was significantly lower. The proportion of patients with anemia (Hb <11 g/dL) was also significantly lower in the FDI group. FDI: ferric derisomaltose; Hb: hemoglobin; TIBC: total iron-binding capacity

Variables	Traditional treatment group (n=38)	FDI group (n=53)	P-value
Days from delivery (days)	34 (27-42)	34 (28-44)	0.88 (U=932)^†^
Hb (g/dL)	11.7 (10.0-13.4)	12.3 (11.1-14.1)	<0.01 (U=534)^†^
ΔHb (from baseline) (g/dL)	4.5 (2.6-6.3)	5.2 (3.8-7.3)	0.01 (U=638)^†^
Reticulocyte (‰)	15.5 (8.3-37.4)	16.1 (5.8-38.9)	0.95 (U=967)^†^
Serum phosphorus (mg/dL)	4.2 (3.2-5.2)	4.1 (2.6-5.2)	0.96 (U=957)^†^
Serum iron (mg/dL)	60 (25-109)	73 (25-144)	<0.01 (U=635)^†^
TIBC (μg/dL)	323 (228-438)	275 (195-371)	<0.01 (U=464)^†^
Ferritin (ng/mL)	79 (17-389)	257 (68-605)	<0.01 (U=160)^†^
Hb <11 g/dL (%)	7 (18.4)	0 (0)	<0.01 (Χ^2^=10.6)^‡^

## Discussion

This retrospective observational study compared the effectiveness of single-dose FDI with that of conventional treatment involving intravenous saccharated ferric oxide and oral sodium ferrous citrate in patients with postpartum anemia caused by hemorrhage. The FDI group showed significantly higher levels of Hb, serum iron, and ferritin at both two weeks and one month postpartum compared to the conventional treatment group.

Notably, the FDI group achieved earlier improvement in anemia. Hb levels were already significantly higher at the two-week mark, and the proportion of patients with anemia (defined as Hb <11 g/dL) was significantly lower in this group. This is likely attributable to the pharmacologic characteristics of FDI, which allows for a single, high-dose intravenous administration that promptly fulfills iron requirements.

At one month postpartum, serum ferritin was also significantly elevated in the FDI group. While ferritin is commonly used as a marker of iron storage, it is important to note that increases in serum ferritin after intravenous iron administration do not necessarily reflect a true increase in long-term iron stores [14]. Intravenously administered iron is taken up by reticuloendothelial macrophages, where it is temporarily stored as intracellular ferritin. Some of this ferritin is then released into circulation, causing a transient increase in serum ferritin levels. This pharmacodynamic response, especially after a high single dose of FDI, may not directly indicate sustainable improvements in iron stores. Long-term follow-up (beyond several months) is necessary to accurately evaluate true iron storage recovery.

The absence of significant differences in reticulocyte counts between the two groups suggests that the degree of erythropoietic stimulation and bone marrow response was comparable. One known side effect of intravenous iron treatment is hypophosphatemia, particularly with ferric carboxymaltose, which has been associated with increased fibroblast growth factor 23 (FGF23) production that suppresses renal phosphate reabsorption [15,16]. FDI, in contrast, has been reported to cause less elevation in FGF23, resulting in a lower risk of hypophosphatemia [17]. In this study, no cases of hypophosphatemia were observed in either group. This may be due not only to the pharmacologic profile of FDI but also to the fact that the study population consisted of relatively young and healthy postpartum women and the follow-up period was short.

The results of this study are consistent with prior international RCTs that demonstrated the efficacy and safety of FDI. While a domestic phase III trial confirmed FDI's non-inferiority to conventional treatment, the present study adds further value by offering real-world clinical data. Notably, the phase III trial in Japan targeted patients with Hb <10 g/dL, while our study focused on those with more severe anemia (Hb <8 g/dL), aligning with FDI's reimbursement indications in Japan. Therefore, the patient selection in this study reflects actual clinical practice, enhancing its practical relevance.

Postpartum iron deficiency anemia should not be regarded as merely an abnormal laboratory finding; it has substantial implications for maternal physical and psychological recovery. Previous studies have shown that persistent anemia can lead to chronic fatigue, impaired concentration, sleep disturbances, and depressive symptoms, all of which may severely disrupt daily activities and caregiving capacity [1-7]. These symptoms are often misattributed to postpartum depression when, in fact, anemia may be a contributing factor. Timely correction of anemia in the early postpartum period is thus critical not only for improving maternal quality of life but also for promoting healthy mother-infant bonding and sustained caregiving.

Strengths of this study include the direct comparison of two widely used therapeutic approaches, namely, single-dose FDI and combined intravenous+oral iron treatment, in a real-world setting. Importantly, FDI was administered as a single inpatient dose without requiring additional outpatient visits, supporting its feasibility and utility in clinical practice. The study also evaluated a wide range of hematologic indicators (Hb, serum iron, TIBC, ferritin, reticulocytes, and serum phosphate) at two weeks and one month postpartum, enabling a comprehensive assessment.

However, the study also has limitations. Being retrospective, treatment assignment was based on physician discretion, which introduces potential selection bias. The significantly higher rate of CS in the conventional treatment group may have influenced outcomes. This discrepancy likely reflects institutional cost constraints under Japan's healthcare system, where the higher price of FDI limits its use in CS cases. Additionally, the study was single-center and involved a limited number of patients, requiring caution when generalizing the results. The follow-up period was limited to one month postpartum, preventing the assessment of long-term outcomes such as sustained iron stores and anemia recurrence. Moreover, although the iron dose should ideally be calculated based on body weight and initial Hb levels [18], this study uniformly administered 1000 mg of FDI without dose individualization. Furthermore, due to the retrospective nature of the study, we could not confirm patient adherence to oral iron therapy after discharge. Although medication was initiated during hospitalization under supervision, variability in outpatient adherence could not be objectively measured and may have influenced long-term hematological outcomes.

Future research should include prospective, multicenter studies with larger sample sizes to more objectively evaluate the long-term efficacy, cost-effectiveness, and patient satisfaction associated with FDI treatment.

## Conclusions

In postpartum patients with Hb levels below 8 g/dL due to hemorrhage, a single intravenous dose of FDI resulted in significantly greater increases in Hb, serum iron, and ferritin levels at both two weeks and one month postpartum compared to conventional treatment combining intravenous and oral iron supplementation. The FDI group also had a significantly lower proportion of anemic patients, and no major safety concerns were observed.

These findings support the real-world utility of FDI, suggesting that it may be a highly effective and practical treatment option, particularly in the early postpartum period when rapid correction of anemia is critical. Further large-scale, prospective studies are warranted to evaluate its long-term efficacy, cost-effectiveness, and impact on maternal and infant outcomes.
